# Muscle-Bone Crosstalk in the Masticatory System: From Biomechanical to Molecular Interactions

**DOI:** 10.3389/fendo.2020.606947

**Published:** 2021-03-01

**Authors:** Sonja Buvinic, Julián Balanta-Melo, Kornelius Kupczik, Walter Vásquez, Carolina Beato, Viviana Toro-Ibacache

**Affiliations:** ^1^ Institute for Research in Dental Sciences, Faculty of Dentistry, Universidad de Chile, Santiago, Chile; ^2^ Center for Exercise, Metabolism and Cancer Studies CEMC2016, Faculty of Medicine, Universidad de Chile, Santiago, Chile; ^3^ School of Dentistry, Faculty of Health, Universidad del Valle, Cali, Colombia; ^4^ Evidence-Based Practice Unit Univalle, Hospital Universitario del Valle, Cali, Colombia; ^5^ Max Planck Weizmann Center for Integrative Archaeology and Anthropology, Max Planck Institute for Evolutionary Anthropology, Leipzig, Germany; ^6^ Department of Human Evolution, Max Planck Institute for Evolutionary Anthropology, Leipzig, Germany

**Keywords:** musculoskeletal system, masticatory muscles, craniofacial bones, paracrine communication, bone biomechanical

## Abstract

The masticatory system is a complex and highly organized group of structures, including craniofacial bones (maxillae and mandible), muscles, teeth, joints, and neurovascular elements. While the musculoskeletal structures of the head and neck are known to have a different embryonic origin, morphology, biomechanical demands, and biochemical characteristics than the trunk and limbs, their particular molecular basis and cell biology have been much less explored. In the last decade, the concept of muscle-bone crosstalk has emerged, comprising both the loads generated during muscle contraction and a biochemical component through soluble molecules. Bone cells embedded in the mineralized tissue respond to the biomechanical input by releasing molecular factors that impact the homeostasis of the attaching skeletal muscle. In the same way, muscle-derived factors act as soluble signals that modulate the remodeling process of the underlying bones. This concept of muscle-bone crosstalk at a molecular level is particularly interesting in the mandible, due to its tight anatomical relationship with one of the biggest and strongest masticatory muscles, the masseter. However, despite the close physical and physiological interaction of both tissues for proper functioning, this topic has been poorly addressed. Here we present one of the most detailed reviews of the literature to date regarding the biomechanical and biochemical interaction between muscles and bones of the masticatory system, both during development and in physiological or pathological remodeling processes. Evidence related to how masticatory function shapes the craniofacial bones is discussed, and a proposal presented that the masticatory muscles and craniofacial bones serve as secretory tissues. We furthermore discuss our current findings of myokines-release from masseter muscle in physiological conditions, during functional adaptation or pathology, and their putative role as bone-modulators in the craniofacial system. Finally, we address the physiological implications of the crosstalk between muscles and bones in the masticatory system, analyzing pathologies or clinical procedures in which the alteration of one of them affects the homeostasis of the other. Unveiling the mechanisms of muscle-bone crosstalk in the masticatory system opens broad possibilities for understanding and treating temporomandibular disorders, which severely impair the quality of life, with a high cost for diagnosis and management.

## Introduction

A strong positive-association between bone mass and muscle mass throughout life has been attributed to their shared function (1–5). This mechano-functional theory has been built upon studies using different approaches. Among these, clinical studies have shown simultaneous decreases in bone and muscle mass when musculoskeletal activity decreases, as in neuronal lesions leading to paralysis, neuromuscular dystrophies, microgravity, immobilizations or prolonged rest (2, 6–8). Likewise, the concomitant loss of both muscle and bone mass in the elderly (sarcopenia and osteoporosis, respectively) leads to a reduction in motility and increases the risk of falls and fractures, heightening morbidity and mortality. How this relationship occurs is not only relevant for basic science; due to the progressive aging of the world population, musculoskeletal disorders are reaching an epidemic status (9, 10). Thus, providing knowledge on the topic can help to the development of prevention and treatment strategies.

Muscle-bone crosstalk, long regarded as exclusively biomechanical, has, over the last decades, been opened to the idea of an additional biochemical component. Thus, muscles and bones are considered secretory tissues capable of releasing soluble molecules to regulate each other (3, 6, 11, 12).

The masticatory system is a highly organized group of craniofacial structures, including bones (maxillae and mandible), teeth, joints, neurovascular elements, and the muscles responsible for moving the mandible. Mandibular movements are required for vital functions such as mastication. These are made possible by the coordinated action of the masticatory muscles (jaw closing and jaw opening) that displace the mandible in a wide range of motions in the tri-dimensional space. That displacement is also guided by the articular surfaces of the temporomandibular joint (TMJ) (13). The biomechanical input from masticatory muscles is not only required for mandibular movement but also for TMJ maintenance (14, 15). The functional and/or structural alterations in one or more of the structures of the TMJ are recognized as temporomandibular disorders (TMDs), grouped by muscular, articular, or developmental conditions (16, 17).

**Figure 1:**
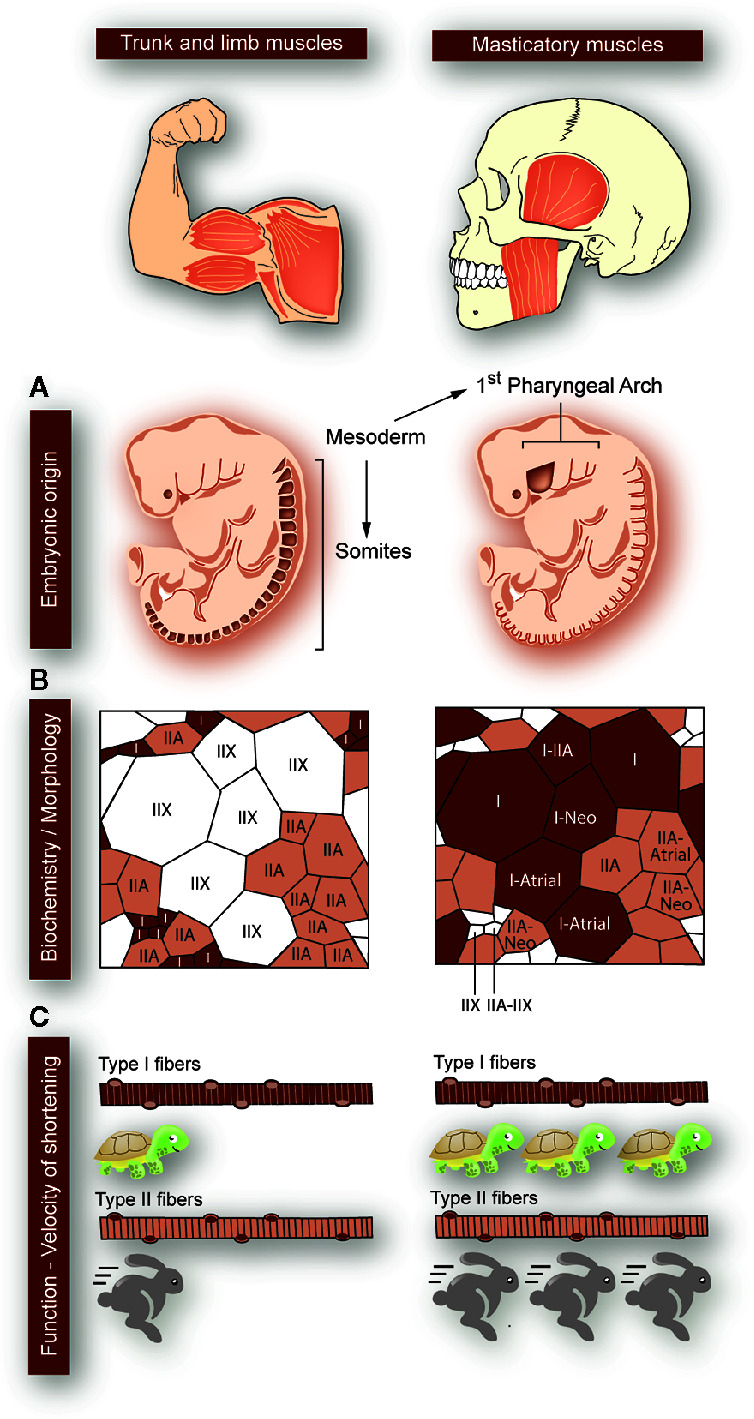
Particularities of masticatory muscles with respect to that of the trunk and limbs. Differences between trunk and limb muscles (left panels) and masticatory muscles (right panels) are depicted, as indicated at the top of the figure. **(A)** While the trunk and limb muscles form from the mesoderm-derived somites, the masticatory muscles are derived from mesodermic-derived cells at the first pharyngeal arch (origin sites colored in dark-brown). **(B)** The trunk and limb muscles express myosin heavy chains (MyHC) type I, IIA, or IIX. Each myofiber expresses a single type of MyHC, and type II fast-fibers have a larger diameter than type I slow-fibers. In masticatory muscles, apart from the classic MyHC types (I, IIA, IIX), the neonatal and cardiac (atrial) types are expressed. There is a large proportion of “hybrid” fibers, simultaneously expressing several MyHCs types. This means that the fibers can have great force-generating properties, with high resistance to fatigue. Additionally, in masticatory muscles, type I fibers are larger in diameter than type II. **(C)** In masticatory muscles, type I myofibers are even 10-fold slower than in trunk and limbs. Moreover, the velocity of shortening of type II myofibers is faster in masticatory muscles as compared to the trunk and limbs ones.

The masticatory system is a highly coordinated machine, where minimal deregulation in one of the components evokes dramatic alterations in the whole system. Because of this, it is an exciting model to study muscle-bone crosstalk. To date, the molecular basis for muscle plasticity or muscle-bone interaction has not been studied in the masticatory system, hindering the development of proper therapies against direct targets in TMDs. Considering that jaw muscles are anatomically and biochemically different to those of the trunk and limb (18–20), it is essential to study them at the cellular and molecular level. Some of the masticatory muscles unique features are: 1) In the embryo, they develop from the mesoderm of the first pharyngeal arch, while the trunk and limb muscles derive from the somites; 2) They express a broader range of myosin heavy chain (MyHC) in adulthood (in addition to type I-IIA-IIX), such as neonatal and cardiac isoforms; 3) They have a high number of hybrid fibers (one fiber expressing several MyHC subtypes), which leads to the development of high force in a fatigue-resistant mode; 4) Their fiber morphology is unusual, with type II fibers smaller in diameter than type I; 5) The velocity of shortening of their type I and type II fibers is even slower and faster, respectively, than their counterparts in the trunk and limbs (**Figure 1**) (20, 21). Moreover, masticatory muscles are highly moldable, depending on genetic and environmental factors (21).

**Figure 2:**
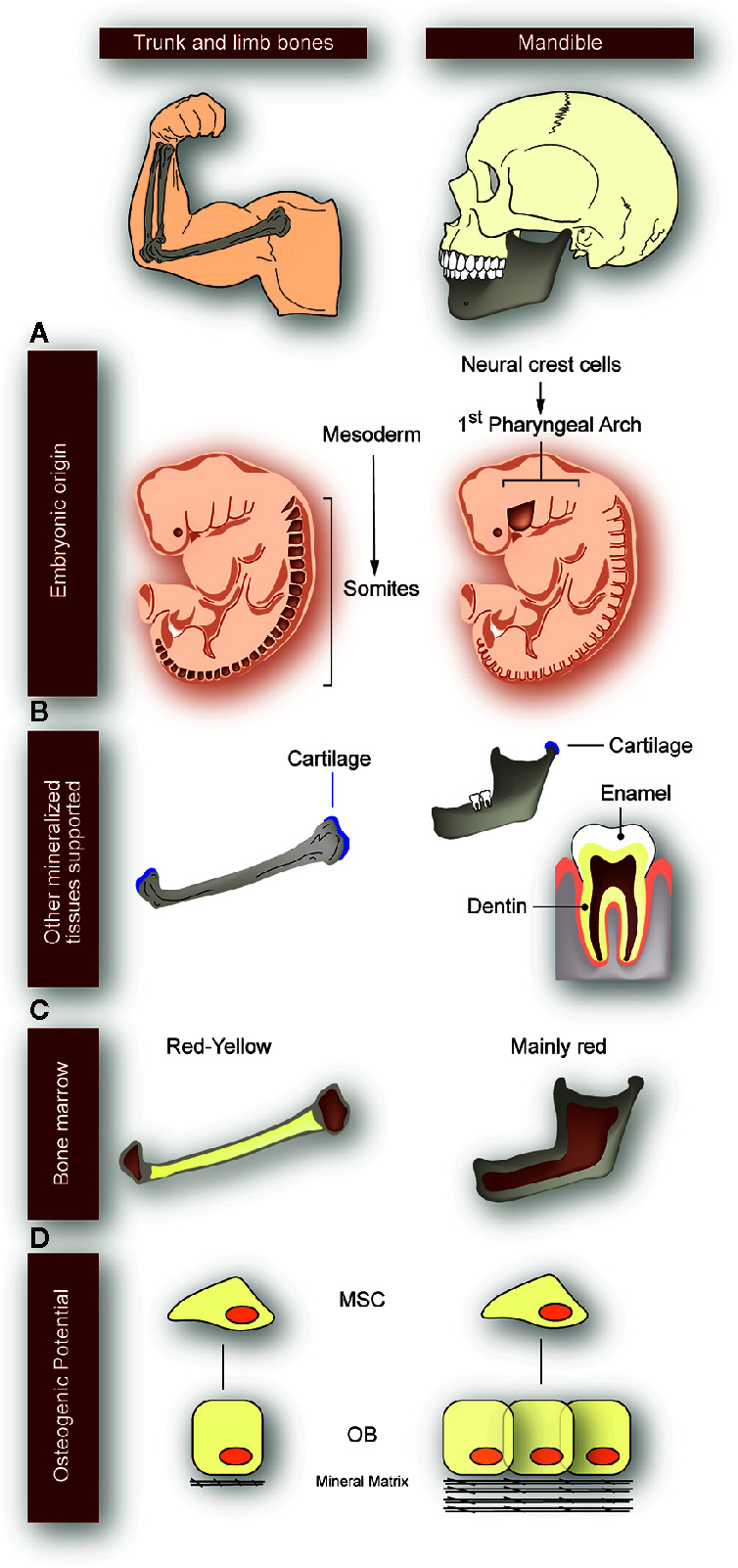
Particularities of the mandibular bone with respect to the trunk and limb bones. Differences between long bones of the appendicular skeleton (left panels) and mandible (right panels) are depicted, as indicated at the top of the figure. **(A)** While long bones derive from embryonic mesoderm, mandibular bone derives from cells of the 1^st^ pharyngeal arch coming from the neural crest (origin sites colored in dark-brown). **(B)** Mandible is the only structure supporting the four main mineralized tissues: bone, cartilage, enamel, and dentin. Instead, long bones only have bone and cartilage. Because jaws support the teeth, they are exposed to additional developmental processes until adulthood and undergo pathologies that are not present in other bones. **(C)** While long bones contain both red and yellow bone marrow, the jaws mainly have red bone marrow. **(D)** Mesenchymal stem cells (MSC) derived from mandible have better osteogenic potential than derived from long bones; they have a higher proliferation rate and mineralization, with an increased regeneration capacity. OB: osteoblasts.

Besides, compared to the postcranial skeleton, the jaws present some unique developmental and morphological features (**Figure 2**). They derive from the embryonic neural crest cells instead of the embryonic mesoderm; they support teeth, which means that they are exposed to additional developmental processes (anatomical and molecular) until young adulthood; they undergo pathologies that are not present in other bones (many of them related to the presence of teeth); as part of the axial skeleton, they contain more red bone marrow than yellow bone marrow; their regeneration capacity is higher than that of the other axial bones; and they are under the constant mechanical stimulus produced by chewing, speech, and swallowing (22–25) (**Figure 2**).

Most of the muscle-bone functional relationship has been addressed through bone biomechanics, i.e. how loading and movement impact bone shape through modeling and remodeling. The cellular processes responsible of this relationship were however not broadly studied. In addition, in the last decade, the molecular crosstalk between bone and muscle has received increasing attention. The present review gathers and organizes for the first time the current evidence of the cross-communication between muscles and bones in the masticatory apparatus, starting from their intimate biomechanical relationship to the current knowledge on molecular cross-talk generated by our own work and the work of other researchers. We propose that, as it occurs with other features of the masticatory apparatus, the muscles and bones of this territory hold a particular biochemical communication through secreted molecules mediating auto/paracrine responses, in particular “myokines” and “osteokines.” Finally, we address how the dysregulation of the masticatory muscles affects the bone component and vice versa, in pathologies, adaptations, or interventions. The latter reinforces the functional interrelation of the components of the masticatory apparatus and challenges to elucidate the molecular bases that mediate this process.

## Biomechanical Interaction Between Muscles and Bones in the Masticatory System; From Function to Shape

Mechanical stimulation, which at a tissue level results in microdeformation of the cells and the extracellular matrix, is an essential factor for bone development and determining bone shape in adults. The mechanostat hypothesis by Harold Frost (26, 27), is still widely accepted among researchers in biomechanics. It proposes that when mechanical stimulation produces bone strains in or above the 1,500-3,000 microstrain range, bone modeling increases bone mass. In comparison, strains below the 100-300 microstrain range activate bone resorption, which reduces unnecessary bone that is metabolically expensive. Low strain magnitudes acting at high frequency are also important in promoting bone formation (28, 29). For this to occur, bone cells responsible for bone deposition and resorption must sense such changes in mechanical stimuli. During muscle contraction and during loading due to e.g., body weight, the deformation of bone tissue, intertrabecular spaces, bone canaliculi and movement of interstitial fluids cause mechanical stimuli that osteocytes sense through mechanoreceptors. This signal is then transduced to different parts within the cell until a target molecule is activated (30).

Although research in this field of mechanoreception and mechanotransduction is still ongoing, some aspects have been elucidated. Among the mechanoreceptors, there are mechanosensitive ion channels that change the polarization status of a cell; integrins that connect the cell membrane with the extracellular matrix and have the inherent capacity to initiate signal transduction; connexins that allow cells in a network to “inform” the others about the mechanical milieu; lipid rafts associated to cytoplasmic second-messengers; and the same cell membrane and primary cilia which during deformation causes conformational changes in molecules that cause the transduction of signals (30–33). During mechanotransduction the cytoskeleton is deformed, which moves organelles and proteins, deforms the nucleus, and activates ion channels and G-protein receptors; in addition, second messengers are activated following the activation of a mechanoreceptor (30, 31). Mechanotransduction ends with the expression of genes and synthesis of proteins such as the receptor activator of nuclear factor kappa-B ligand (RANKL), sclerostin, osteopontin, and fibroblast growth factor 23, among others relevant for bone homeostasis (30, 33).

**Figure 3:**
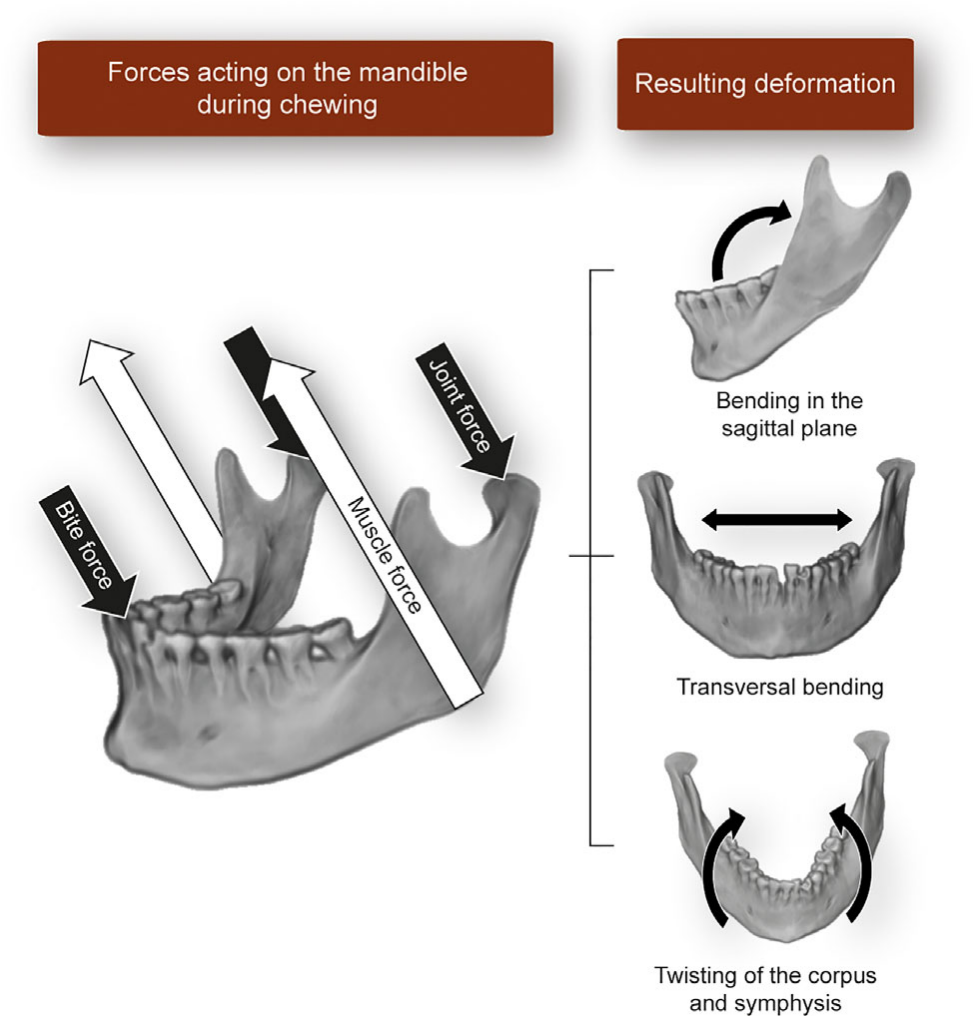
Schematic representation of the forces acting on the mandible during static biting and the resulting bone deformation patterns described in the literature. Image built using a three-dimensional reconstruction of CT-data from an individual in Toro-Ibacache et al. (36).

The masticatory apparatus produces loads of variable magnitude and high frequency on the teeth and jaws. Unlike in the appendicular skeleton, the loads cause complex patterns of bone deformation during normal function. These cause bone modeling and remodeling, which ultimately shapes the adult form of the jaw to a mechanically fit morphology. These loads are produced directly by tension in the entheses, but perhaps more markedly, by microdeformation of the entire structure as a result of the different force vectors acting on it. During the power stroke of mastication, maximal muscle activity and bone strain occur. Forces acting on the jaw during the power stroke can be represented in a simplified manner using lever mechanics, where the TMJ acts as the fulcrum, the distance of muscle insertion to the TMJ is the force arm, and the biting force is the resistance arm. The more anterior the biting point, the lower the resulting biting force, and vice versa. In a frontal plane, a more laterally placed biting point (e.g., at the posterior teeth of animals with wide palates like humans) is close to the TMJ, reducing the length of the resistance arm and increasing bite force. The logical conclusion is that biting in posterior teeth is more efficient in terms of the use of muscle force. A more detailed review on the mechanics of biting in humans can be found in Hylander (34) and Lieberman (35). In this simplified model, not only the biting point and the entheses (at the cranium and mandible) are loaded, but also the TMJ surfaces. The applied muscle force magnitude decomposes at the biting point and the TMJ. Thus, if a large muscle force is generated during contraction, a large bite force reduces the reaction force at the TMJ. The resulting forces produced during biting cause the deformation of the jaw (**Figure 3**). Due to its simpler anatomy compared to the maxilla, the mandible has undergone most of the studies in this regard. Using experimental and theoretical approaches in humans and non-human primates, it has been shown that the mandible deforms in three main patterns: bending of the sagittal plane, transversal bending (also called “wishboning”), and twisting of the corpus and symphysis (37–40) (**Figure 3**). Studies by Daegling (37) and Fukase (41) analyzing the morphology of the mandible in macaques and humans, respectively, concluded that a thick cortical bone is located in the anterior part of the mandible and towards the posterior end of the corpus, precisely where strains during biting are the largest.

The study of the primate cranium has presented a comparable level of evidence regarding the impact of biting forces on bone strain and, thus, morphology. Bromage found that the orientation of collagen fibers in the circumorbital region of macaques follows the directions of strains produced on it during biting (42). These strains, however, although enough to have an impact at the tissue level, would not be the cause of presence of the supraorbital torus in these animals (43). In a series of experimental and observational studies, Toro-Ibacache et al. showed that during incisor and molar biting in humans, the most strained areas of the face are the alveolar process in relation to the loaded tooth and the frontal and zygomatic processes of the maxilla (44–46). These areas at the same time are formed by dense bone that forms the cranial buttresses and also show evidence of bone deposition (47). The deformation pattern in the human cranium does not vary much from that of non-human primates, consisting of a ventral bending of the anterior part of the maxilla during incisor biting (45, 48), and compression of the lateral aspect of the orbit during molar biting (45, 49).

Considering the evidence above, it is then logical to assume that a modification of masticatory forces also affects skull form. Experimental work in animal models has shown that the impairment of masticatory muscle activity and/or softening food consistency leads to a reduction in the thickness of the trabecular bone of the condylar process (50, 51), to a reorganization of it (51, 52), and to a gracilization in the form of the entire skull (53, 54). Observational studies in humans agree with these results. Raphael et al. (55) showed in women with TMJ disorders that those who had received intramuscular injections of botulinum toxin had a decreased bone density of the condylar process in compared to women who did not receive the injections. Overloading of the TMJ could also lead to degeneration of the joint components (56, 57). Egli et al. found in people affected by Duchenne muscular dystrophy that they progressively developed malocclusions, which are associated with lowering bite forces and a detriment of the masticatory function (58). Moreover, it has been proposed that the diet of the modern, urban populations, based mainly on highly extraorally processed food items, is the cause of a reduction in jaw size that results in dental malocclusions in modern humans which are not found in their ancestors (59–61). In addition, dental malocclusions reduce masticatory efficiency (62), and altered maxillomandibular relationships are at the same time associated to broad ranges of craniofacial shape variation (36, 63, 64).

From a developmental perspective, an optimal masticatory function should act by canalizing craniofacial form. However, this does not seem to be the rule in modern humans. Although it is possible to find a relationship between the intensity of masticatory activity and the shape of the craniofacial skeleton, this seems relatively modest, increasing only when functional limits are reached, i.e. when either very high/low/infrequent force magnitudes or altered force vectors are produced. In this regard, Toro-Ibacache et al. found that modifying the patterns of masticatory muscle activity (i.e. the relative force produced by each jaw-closing muscle during biting) changes the peak strain magnitudes, but not where these are located; only large, asymmetric modifications were able to change the location of TMJ peak strains from the balancing to the working side (45). Moreover, the same author described a weak covariation between masticatory muscle force and craniofacial shape in humans (46) and found differences in mandibular and cranial shape only among groups of individuals with marked differences in diet consistency/dental occlusion pattern (36, 63). Shape covariation between the upper and lower jaws/dental arches is also lower in humans when compared with other animals (65), which has been associated with the comparatively lower mechanical and kinetic constraints underwent by humans during the normal masticatory function. Conversely, stronger covariation has been found in individuals with malocclusions when compared with those with normal occlusion (64, 66). Taken together, these antecedents support the idea that in humans, there has been a reduction in the constraining role of the masticatory function on the shape of the craniofacial skeleton. This means that under physiological conditions that do not involve intense nor too low masticatory forces, the human cranium displays a broad range of morphologies, which may be the result of other, non-mechanical factors. However, outside these functional limits, there are morphological consequences on craniofacial bones, as seen in the aforementioned studies with congenital and induced muscle paralysis and dental malocclusions. How this can be of use in the clinical context has been in part explored; changes in how the musculoskeletal system works is the basis of orthopedics and other related disciplines, such as orthodontics and management of TMJ disorders. However, achieving predictable, long-term results is sometimes challenging. For example, some malocclusions relapse after the end of orthodontic treatment, and some individuals do not respond to the orthopedic management of TMJ disorders. At the same time, the use of extreme functional settings or the use of large external forces to induce bone changes can also cause tissue damage. Thus, how to achieve a fine-tuning of the relationship between forces and bone (and articular tissues) morphology is yet to be understood.

In conclusion, chewing generates forces that cause the deformation of the skeleton. This deformation is sensed at the cellular level, eliciting a response that can result in bone resorption or deposition. These processes modify the shape of the loaded bone, turning it into a structure able to withstand the new loading scenario. This relationship, however, is not always linear. In humans, whose masticatory activity is less intense compared to that of other mammals, the shape of the craniofacial skeleton is remarkably variable, and it does not necessarily correlate to masticatory function parameters. However, under extreme functional situations, the form of the jaws is more prone to reflect the loading scenario. Controlling this non-linear relationship between form and function could be key in achieving predictable, long-term results in clinical situations where functional or externally applied forces are the therapeutic tools.

## Masticatory Muscles and Bones as Secretory Tissues. Muscle-Bone Interaction Through Signaling Molecules

### Muscle-Bone Crosstalk; Looking Beyond Mechanics

The relationship between muscles and bones in health and disease has been mainly considered as a mechanical process in which bone provides an attachment site for muscles and muscles apply load to bone (67). It is known that bone adjusts its mass and architecture to changes in mechanical load, so it is strongly influenced by muscle contraction (68). In recent years, the idea has emerged that beyond this mechanical coupling of muscle and bones, there is a biochemical crosstalk through secreted molecules (11). In effect, both muscle and bone produce factors that circulate and act on distant tissues, the classical definition of an endocrine signal. Therefore, molecular communication with its adjacent tissue is even more likely to occur. Understanding this apparent biochemical coupling between muscles and bones is an exciting new avenue of research (2, 3, 6, 69). Muscles and bones have been recently defined as endocrine organs, secreting “myokines” and “osteokines.” respectively. These molecules are secreted after a wide range of stimuli and run autocrine, paracrine, and endocrine effects in several tissues. A recent review by Kirk et al. summarizes the current knowledge about molecules involved in musculoskeletal communication, including not only myokines and osteokines, but also adipokines (12). The list of currently defined myokines includes myostatin, interleukin (IL)-5,6,7,8,15, brain-derived neurotrophic factor (BDNF), Irisin, Insulin-Like Growth Factor (IGF), β-aminoisobutyric acid (BAIBA), matrix metalloproteinase-2, and Fibroblast Growth Factor (FGF)-2, which mediate the crosstalk between skeletal muscles and adipose tissues, blood vessels, central nervous system, and/or bone (3, 6, 70–72). Muscle-derived exosomes and miRNAs have been found in the circulation and influenced by exercise and disease, but their paracrine/endocrine role on other tissues has been not well-established (73, 74). Actually, efforts are directed towards muscle secretome elucidation (75, 76). Likewise, bone cells, which historically have been considered a target of the endocrine system, have been described in recent years as secretory entities of signaling molecules for controlling local or long-distance processes (77–79). Molecules suggested as osteokines include Osteocalcin (80), Sclerostin (81), Prostaglandin-E2 (PGE2) (81), Fibroblast Growth Factor 23 (FGF-23) (82), Transforming Growth Factor β (TGF-β) (83), RANKL (84, 85), and Wnt3a (81, 86)

The intimal muscle-bone relation is strongly evidenced by the fact that in open fractures, if muscle injury is also extensive, or if muscle atrophy develops, healing of the fracture is significantly impaired (11, 87, 88). In contrast, when the fracture area is covered with muscle flaps, even without tendon attachment, bone repair is significantly improved (88). This reinforces the communication between muscles and bones through soluble molecules, complementary to signaling by mechanical loading. In a mouse model of deep penetrating bone fracture and muscle injury, the exogenous administration of recombinant myostatin significantly reduced bone callus formation, while increasing fibrous tissue in skeletal muscle (87). Furthermore, assays using conditioned media coming from C2C12 cultured myotubes demonstrated that skeletal muscle-secreted factors protect the osteocytes against apoptosis evoked by glucocorticoids, by activating the β-catenin pathway (89). In the opposite way, conditioned media derived from osteocytes evokes calcium transients and myogenesis of a C2C12 cell line, mimicked by the bone-released factor prostaglandin E2 (PGE2) (81).

### Biochemical Muscle-Bone Crosstalk in the Masticatory System—From *In Vitro* to Preclinical Evidence

As previously described, the muscle-bone relationship in the masticatory system has been mostly studied from a biomechanical perspective. However, differentiating the mechanical component and the biochemical signaling by secreted molecules is not straightforward. Nowadays, there are no studies available that address biochemical crosstalk between muscles and bones in the masticatory system. However, several molecules described as myokines or osteokines in the musculoskeletal system of the trunk and limbs have been reported acting in the masticatory region, allowing to propose their putative role in bone-muscle communication.

A common criticism of the idea of biochemical communication between muscles and bones is that the molecules released from one of them must pass multiple tissue barriers to move from one tissue to another. The presence of physical barriers such as endomysium, perimysium, and epimysium in muscle and periosteum in bone is always mentioned. However, it has been demonstrated the presence of vessels coming from muscle in bone, in direct relation to osteocytes (3), which would allow a rapid endocrine effect between them. In particular, the injection of bone marrow mononuclear cells into rat masseter muscle has been shown to help repair bone after mid-palate expansion procedures (90). Therefore, if cell migration between the masseter and the palate occurs, communication *via* molecules is highly probable.

Next, we list several molecules well-described as muscle-bone interactors in the trunk and limbs and analyze the background suggesting their involvement in the masticatory apparatus.

#### Myostatin

Myostatin (GDF-8) is a member of the TGF-superfamily. It is released by muscle cells and acts as an autocrine negative regulator of muscle mass (91). An increase in myostatin levels is related to conditions of skeletal muscle injury, disuse, or sarcopenia (92, 93), and limits the bone formation/resorption index (94). In contrast, reduction of myostatin expression by using genetic approaches or pharmacological inhibitors highly increases skeletal muscle mass, bone formation, and bone regeneration (95–98). The effect of myostatin on bone remodeling has historically been attributed to its direct effect on muscles and their biomechanical role on the skeleton. However, it is currently known that myostatin has a direct impact on bone cells, such as the acceleration of osteoclasts formation evoked by RANKL (99) and the inhibition of osteoblasts differentiation by controlling the content of the exosomes derived from osteocytes (74). This is why myostatin has emerged as one of the candidates in muscle-bone crosstalk. Knockout mice lacking myostatin, called **“**mighty mice,**”** have higher morphologic dimensions of the mandibular condylar process, corpus, and symphysis (100). Moreover, they have increased mineralization at the corpus (101), as well as in the condylar subchondral bone and mandible neck (102). Myostatin-deficient mice have longer and **“**rocker-shaped**”** mandibles, with shorter and wider crania compared to controls (103).

##### Insulin-Like Growth Factor 1

**Figure 4:**
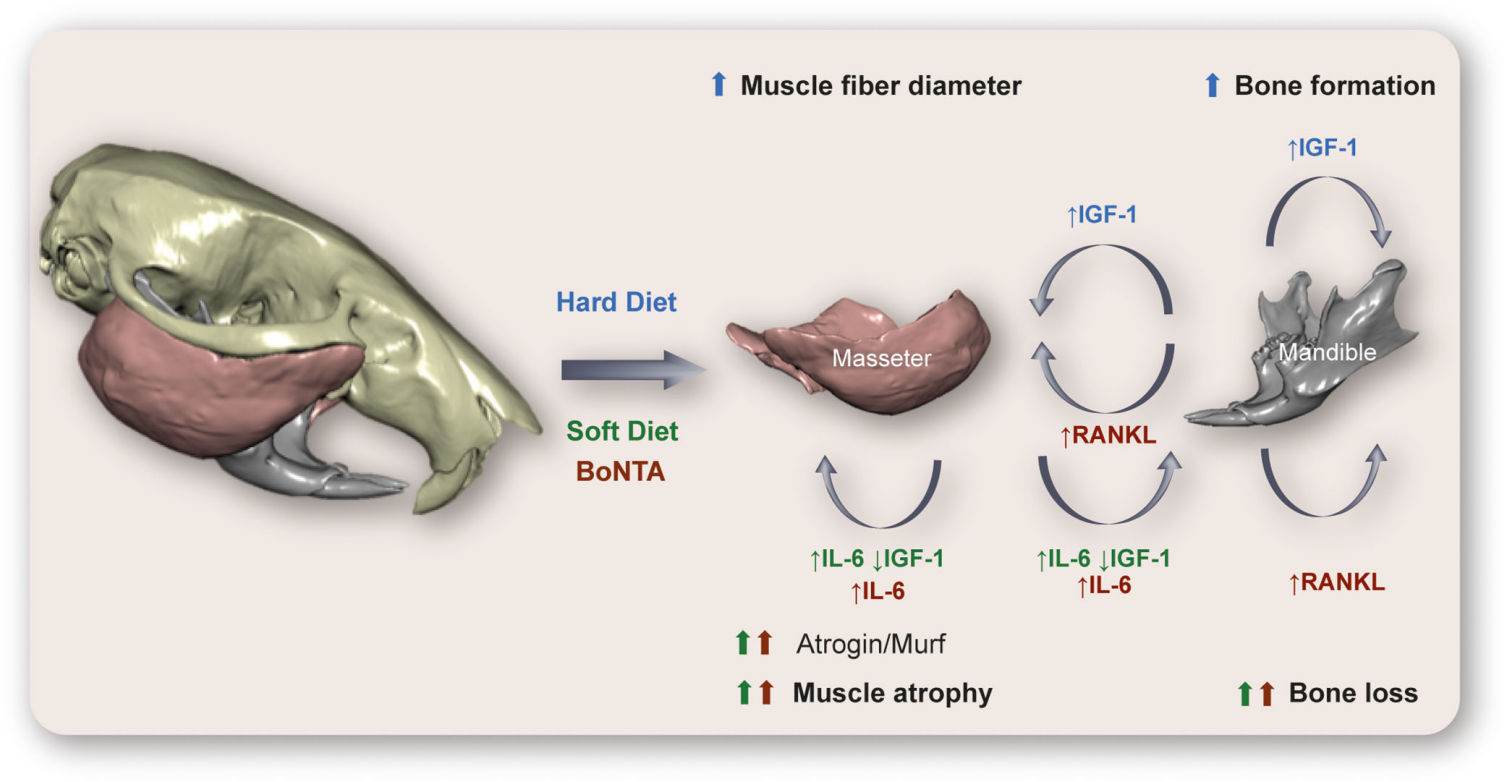
Hypothetical model of cross-communication between muscles and bones at the murine masticatory system. Here we relate in a graphic outline the main changes described in rat/mouse models subjected to a reduction (Soft Diet) or an increase (Hard Diet) in diet consistency, as well as those described after paralysis of the masseter muscle by injection of botulinum toxin type A (BoNTA). In the hypofunctional models (Soft-diet, BoNTA), an increase in interleukin-6 (IL-6) expression and release, as well as a reduction in insulin-like growth factor 1 (IGF-1) in masseter muscle could mediate the muscle atrophy and bone loss, together with the reduced mechanical stimulation. In addition, the increased levels of RANK ligand (RANKL) in mandibular condyle after BoNTA injection could mediate both the osteoclastogenesis leading to bone loss and the muscle atrophy observed. On the other hand, consumption of a hard diet evokes an increase in IGF-1 expression in mandibular osteocytes, which could act as an anabolic factor in muscle and bone, leading to increased muscle mass and bone formation described in this model. *Technical information*: 3D rendering of murine skull, mandible, and masseter muscles corresponds to PTA contrast-enhanced high-resolution microCT data taken at the Max Planck Institute for Evolutionary Anthropology (Leipzig, Germany). Skull and mandible segmented with Avizo 9.2 (Thermo Scientific™, USA); masseter muscles segmented with the Biomedical Segmentation App (Biomedisa) (128). 3D rendering of hard and soft tissues performed with DRAGONFLY 4.1 (Object Research Systems, Canada). Image built using data from an individual in Balanta-Melo et al. (129).

IGF-1 is mainly produced in the liver, but it is also expressed in skeletal muscle and highly increases after exercise (104). In addition, IGF-1 is the main growth factor in the bone matrix (105); it is expressed by osteocytes and upregulated in response to mechanical loading (106). It is also well known that IGF-1 is a relevant anabolic factor in muscle and bone and has been proposed as a potential muscle- bone crosstalk molecule (107). Several functional changes in skeletal muscle, such as unloading, overloading, or denervation, modify the expression of proteins of the IGF-1 signaling pathway, which relates to changes in muscle fiber type (104, 108). When rats are fed with a soft diet immediately after weaning, the mRNA levels for IGF-1, IGF-2, IGF receptor (IGFR) 2, IGF binding proteins (IGFBP) four and six in masseter muscle are reduced (109). Furthermore, in the masseter muscles of mice feeding a soft diet after weaning, there is a reduction in IGF-1, IGF-2, and IGFBP5 expression (110). In parallel, murine models of masticatory reduction through soft diet consumption show alterations in morphology and molecular markers in masseter muscle and mandible (**Table 1**, **Figure 4**). A decrease in masseter muscle activity has been reported (114, 115). Furthermore, a reduction in masseter muscle mass and fibers diameter have been demonstrated in rats (110, 117) and mice (111, 118, 124) after soft-diet consumption. MyHCIIB (fast glycolytic) expression levels are increased in a 580%, and MyHCIIA (fast-oxidative) mRNA levels are reduced in a 70% in rats fed with a soft diet (109), consistent with observed in all muscle disuse models. It has been established that the increased expression of genes related to hypercatabolism works as a molecular marker of muscle atrophy. Of these, the most studied are the ubiquitin ligases muscle RING finger 1 (Murf1) and muscle atrophy F-box (MAFbx or Atrogin), relevant components of the ATP-dependent ubiquitin-proteasome pathway (130). We have recently reported an increase in atrophy markers Atrogin and Murf in the masseter muscle of adult mice, as early as 2 days after start eating a soft-diet (4- and 20-fold increase, respectively). After 30 days of consuming the soft diet, the levels of Atrogin and Murf expression were increased by 35- and 150-fold, respectively, compared with mice eating regular pellets (118, 124). In mice and rats, the soft-diet consumption modifies both mandible and condylar morphology, by reducing mandible ramus height and robustness and condylar width (54, 57, 113, 115, 117). A reduction in bone volume fraction of the mandibular condyle and masseter muscle attachment sites have been observed, as well as a reduction in articular cartilage thickness (112, 115, 116, 131).

**Table 1:** Summary of adaptations in the masseter muscle and mandible in murine models of masticatory hypofunction.

A. Soft diet consumption					
Reference	Effect on masseter			Effect on mandible	Myokines/Osteokines
Vilmann et al (111).	↓ Fibers diameter				
Saito et al (109).	↓ Type IIA fibers ↑ Type IIB and type IIX fibers				↓ IGF-1 and IGF-2 expression
Urushiyama et al (110).	↓ Muscle mass ↓ Fibers diameter				↓ IGF-2 expression
Tanaka et al (112).				↓ Degree of mineralization in the trabecular bone of the mandibular condylar process	
Odman et al (113),				↓ Posterior height of the mandibular corpus and the height of the angular process	
Kawai et al (114).	↓ Muscle activity ↓ Type IIA fibers ↑ Type IIB fibers ↓ Cross-sectional area of type IIB and IIX fibers				
Hichijo et al (115).	↓ Muscle activity			↓ Condylar articular cartilage thickness ↓ Mandibular ramus height	
Hichijo et al (116).				↓ BV/TV of the mandibular condyle and the masseter attachment sites	
Spassov et al (54).	↓ Muscle mass			Horizontally-oriented coronoid process and smaller mandibular condylar process articular surface	
Shi et al (117).	↓ Muscle mass			↓ Tb.Th and Tb.N of the mandibular condylar process ↓ Condylar articular cartilage thickness	
Rojas-Beato et al (118).	↓ Muscle mass ↑ Atrophy markers (Atrogin/MuRF)				↑ IL-6 expression and synthesis
**B. Masseter muscle intervention with BoNTA**					
**Reference**	**Animal (age)**		**Effect on masseter**	**Effect on mandible**	**Myokines/Osteokines**
Tsai et al (119).	Male rats (4 wks)		↓ Muscle mass	↓ Total mandibular length	
Tsai et al (120).	Male rats (8 wks)		↓ Muscle mass	↓ Mandible dimensions, BMD, Cortical Bone Thickness and Trabecular Bone Area to Total Bone Surface	
Tsai et al (121).	Male rats (10 wks)		↓ Muscle activity (transient, up to 4 wks)		
Kün-Darbois et al (122).	Male rats (18 wks)			↓ B.Ar/T.Ar of the alveolar and the mandibular condylar process	
Dutra et al (123).	Female mice (5 wks)			↓ BV/TV, Tb.Th, width and tissue density of the mandibular condylar process ↑ Apoptosis and ↓ proliferation in both subchondral bone and articular cartilage of the mandibular condylar process	
Shi et al (117).	Female rats (5 wks)		↓ Muscle mass	↓ BV/TV, Tb.Th, Tb.N, width and length of the mandibular condylar process ↓ Condylar articular cartilage thickness ↑ Tb.Sp of the mandibular condylar process	
Balanta-Melo et al (50).	Male mice (8 wks)		↓ Muscle mass ↓ Fibers diameter ↑ Atrophy markers (Atrogin/MuRF) and Myogenin mRNA expression	↓ B.Ar/T.Ar and Tb.Th of the mandibular condyle	↑ RANKL mRNA expression in the mandibular condylar process
Balanta-Melo 2018b (124).	Male mice (8 wks)				↑ IL-6 expression
Balanta-Melo et al (51).	Male mice (8 wks)		↓ Muscle mass	↓ BV/TV, Tb.Th and shape changes of the mandibular condylar process	
Balanta-Melo et al (125).	Male mice (8 wks)			↓ BV/TV and Tb.Th in the middle portion of the mandibular condylar process ↓ BMD of the mandibular condylar process	
Dutra and Yadav (126).	Female mice (6 wks)			↓ BV/TV and articular cartilage thickness of the mandibular condylar process (not transient, up to 8 wks) ↑ Apoptosis in articular cartilage of the mandibular condylar process	
Vásquez et al (127).	Male mice (8 wks)		↓ Muscle mass ↓ Fibers diameter ↑ Atrophy markers (Atrogin/MuRF) and Myogenin mRNA expression		↑ IL-6 expression

Evidence regarding morphological and biochemical changes in the masseter muscle and/or the mandible in murine models of soft-diet consumption (A) or masseter paralysis evoked by BoNTA injection (B) are listed. Changes in expression of molecules classically described as myokines or osteokines are highlighted.

wks, weeks; mo, months; BV/TV, bone volume fraction; Tb.Th, trabecular thickness; Tb.N, trabecular number; Tb.Sp, trabecular space; B.Ar/T.Ar, bone area per tissue area; BMD, bone mineral density; IGF, Insulin Growth Factor.

On the other hand, it has been recently demonstrated that a mouse model of increased mastication by hard-diet consumption shows raised levels of IGF-1 and a decrease in sclerostin expression in osteocytes. In this model, an increase in bone formation at the enthesis of the masseter muscle was observed (132).

Then, the IGF-1 signaling either decreases or increases after hypo- or hyperfunction of the masticatory system, respectively. IGF-1 is expressed by both the masseter muscle fibers and mandibular osteocytes, which makes it a strong candidate for mediating muscle-bone crosstalk in this territory (**Figure 4**).

##### Interleukin-6 (IL-6)

IL-6 family of cytokines comprises ten members, such as IL-6, IL-11, leukemia inhibitor factor (LIF), and oncostatin M (OSM). IL-6 synthesis and release have been historically related to immune cells, for mediating B- and T-cells development with a pro-inflammatory role (133). However, nowadays, it is known that there are several sources of IL-6, such as epithelial cells, fibroblasts, osteoblasts, synovial cells, cancer cells, and skeletal muscle fibers, leading to either pro- or anti-inflammatory events (134, 135). IL-6 is a 26 kDa glycopeptide that binds a specific IL-6 receptor (IL-6R, either membrane-associated or soluble (sIL-6R)) (134). IL-6 was the first molecule defined as **“**myokine**”** when Pedersen and colleagues demonstrated a link between IL-6 and exercise (136). IL-6 is a myokine released from skeletal muscles during exercise, with a plethora of physiological effects in autocrine, paracrine and endocrine ways (135, 137, 138). The magnitude of the plasma-IL-6 increase depends on the exercise duration, intensity, and muscle mass involved. Plasma levels of IL-6 increase up to 100-fold after exercise, without any sign of muscle damage, nor an associated inflammatory response. Furthermore, the concentration of IL-6 in the interstice of the exercised muscles, measured by microdialysis, is up to 100 times higher than the plasma concentration, suggesting a possible local role (139).

IL-6, in an autocrine-loop, improves insulin sensitivity in skeletal muscle cells for increasing glucose uptake (140, 141). In addition, IL-6 produced in response to strenuous and prolonged exercises increases satellite cells proliferation, leading to regeneration of damaged muscle myofibers and hypertrophy (142, 143). However, a pivotal role of IL-6 in skeletal muscle has been proposed, being related to both skeletal muscle renewal and wasting. Under some pathological conditions, IL-6 leads to muscular atrophy. During cachexia [muscle wasting associated with underlying chronic diseases such as cancer, chronic heart failure, and chronic kidney disease (144)] it has been proposed that increased IL-6 plasma levels mediate proteolysis at the skeletal muscle in patients. In a mice model of cancer-evoked cachexia, activation of the IL-6 signaling pathway has been demonstrated in skeletal muscles, increasing both phosphorylation and nuclear localization of STAT3 (145). Moreover, pharmacological or molecular blockade of the IL-6/STAT3 signaling pathway prevents tumor-induced muscle atrophy in mice (146). Either IL-6 infusion in wild type mice or the transgenic mouse models for IL-6 overexpression, evoke muscle atrophy by reducing protein synthesis and promoting catabolic pathways (147–149). Several pharmacological therapies targeting the IL-6 signaling pathway, mainly by using anti-IL-6 or anti-IL-6R antibodies or blockers, have had preventive effects in cancer-evoked cachexia (150, 151), restored muscle function in a mouse model of muscular dystrophy (152), and ameliorated muscle atrophy induced by tail suspension in mice (153).

In bone physiology, IL-6 also shows a dual role. IL-6 influences both osteoclasts and osteoblasts differentiation through contradictory mechanisms. It sustains bone formation, but it also drives bone loss in various osteolytic pathologies (134). Osteoblasts express low levels of IL-6R; therefore, sIL6R is required for maximum IL-6 effects. IL-6 family members increase osteoblasts markers expression and matrix mineralization nodules, all mediated through the STAT3 activation (154, 155). In contrast, IL-6 type cytokines (IL-6, IL-11, LIF, and OSM) inhibit bone formation *in vitro*, with potent pro-apoptotic effects, all mediated by PKCδ and ERK1/2 pathways. IL-6 clearly stimulates the osteoblastic production of RANKL and PGE2, both involved in differentiation and activation of osteoclasts; this has been described as the critical event leading to pro-resorption action evoked by IL-6, and is mediated by STAT3 signaling (156–158). In contrast, other research groups have described that IL-6 inhibits osteoclast formation and bone resorption in pre-osteoclasts primary cultures or cell lines (159, 160). By using genetic strategies, it has been demonstrated that the IL-6 deficient mice have increased bone formation, whereas IL-6 overexpression showed a decrease in osteoblasts and osteoid, but also in osteoclasts and bone resorption. Then, it has been proposed that IL-6 could contribute to bone turnover *in vivo* (134). An essential role of IL-6 in osteoarticular pathologies has been established. IL6-/- mice are protected from joint inflammation and destruction in a mouse model of arthritis, and from bone loss evoked by estrogen depletion. Inhibition of IL-6R with the drug Tocilizumab improves the clinical response and suppresses the biochemical markers of osteoclast-mediated bone destruction in patients with rheumatoid arthritis (161, 162). In contrast, IL-6 stimulates fracture healing and bone resistance (163). All of this data suggests that IL-6 can lead to bone formation or resorption, depending on its pathophysiological context. The role of IL-6 in bone turnover is then indisputable; however, it is not easy to directly associate it to a specific effect, due to it appears as a double-edged sword.

At the masticatory system, the role of IL-6 in muscle homeostasis has been demonstrated. An increase in masticatory activity in a mice model of restrained/gnawing raises IL-6 mRNA and protein levels in the masseter muscle. The increase in IL-6 production and release is dependent on a previous rise in IL1α-β, and then promotes the glucose uptake in the masseter muscle. Authors suggest that a highly coordinated loop happens, where masseter muscle activity releases some myokine that “calls to” IL-1 positive cells around blood vessels; then, IL-1 evokes IL6 expression and release from masseter muscle, improving the glucose homeostasis and muscle performance and preventing muscle fatiguability (164). Ono et al. also reported an increase in IL-6 in rat masseter muscle when stimulated electrically *in situ* (100* Hz* for 200 ms, 800 ms between stimulations, 10-30-60 min total stimulation time) (165). In masseter muscles isolated after the electrical stimulation protocol, they observed a 3-fold increase in IL-6 mRNA levels, with no changes in IL-1 β mRNA levels. These authors propose that considering that IL-1 β is a well-known pro-inflammatory cytokine, the increase in IL-6 in masseter muscle would not respond to inflammatory infiltration, but a local synthesis in muscle fibers. When carrageenan was directly injected in rat masseter muscles, which is an inductor of local acute inflammation, both IL-6 and IL-1 β mRNA levels increased. When the electrical stimulation was performed after muscle contraction blockade with dantrolene, the increase in IL-6 mRNA was blocked, suggesting that muscle contraction is relevant to evoke IL-6 expression. The authors reinforce the idea that masseter muscle contraction stimulates IL-6 expression, independent on inflammation processes (165).

Some of us have recently demonstrated that electrical stimulation of isolated masseter muscle *in vitro* (20 Hz, 270 pulses, 0.3 ms each), resembling motoneuron stimulation, evokes an increase in IL-6 mRNA expression, as well as IL-6 protein synthesis and release (118). Thus, masseter muscle synthesizes and releases IL-6 during activity. However, as previously described, IL-6 has a pivotal role, as it has either anabolic or catabolic effects in the musculoskeletal system. We have recently demonstrated basal increases in IL-6 production and secretion in mouse models of masseter muscle atrophy. In the previously described model of adult mice consuming a soft diet, a 2-fold increase in IL-6 mRNA was observed in the masseter muscle, as early as 2 days after soft-feeding. Two weeks later, resting levels of IL-6 mRNA and protein increased 12-fold and 2-fold, respectively, compared with mice eating regular pellets (118, 124, 166, 167). Thus, IL-6 is highly overexpressed in a mouse model of masseter muscle atrophy by underuse. We also developed a mouse model of unilateral injection of Botulinum Toxin type A (BoNTA) in the masseter muscle, specifically to address putative alterations in the associated bone (mandibular condylar process) evoked by muscle paralysis. This is highly relevant in dentistry because BoNTA is used as an off-label therapeutic tool for the management of several TMDs (125, 168). In adult mice, we injected the right masseter with 0.2U/10 µl BoNTA, and the left masseter with saline solution. As early as 7 days after the intervention, an increase in molecular markers of muscle atrophy (Atrogin and Murf1) was observed, with histological signs of atrophy after 14 d (50, 127, 167, 169). A reduction in masseter muscle activity, muscle mass and fibers diameter have also been observed after BoNTA injection in masseter muscles of rats and mice (50, 117, 119–121, 170). Interestingly, we demonstrated an increase in IL-6 mRNA levels in muscles as early as 2 days after BoNTA injection (50, 127, 167, 169). Just 2 days after BoNTA injection in masseter muscle, a significant increase in a molecular marker of bone resorption (RANKL) was also observed in the ipsilateral mandibular condylar process. Two weeks after BoNTA injection, qualitative bone loss was detected in the right mandibular condyle (BoNTA-side), with highly impaired bone parameters detected by microcomputed tomography (µCT). In contrast, contralateral saline-injected masseter muscle and its adjacent condylar process remained as healthy as that in control untreated mice (51, 125). Several other authors have observed severe damages in mandibular morphology and microstructure after BoNTA injection in masseter muscles of murine, with high impact in the articular cartilage and subchondral bone (117, 119, 120, 122, 123, 126, 170). Then, BoNTA injection evokes both muscle atrophy and bone loss at the mandibular condylar process (as summarized in **Table 1** and **Figure 4**). We are currently studying the putative role of IL-6 myokine in both muscle atrophy and bone loss evoked by BoNTA injection. Taken together, these results support the idea that IL-6 is released from masseter muscle either during activity and during paralysis/atrophy, reinforcing its dual role in physiology and pathology of the musculoskeletal system.

Interestingly, IL-6 level at the synovial fluid has been widely associated with TMD (171). IL-6 level is undetectable in synovial fluid from healthy controls (172, 173), but it is increased in that from patients with chronic TMD (174). Moreover, in TMD patients, IL-6 level at the synovial fluid is significantly higher in the joints with bony changes in the condylar processes than when these are not affected (175). Then, IL-6 could be associated with bone remodeling during TMDs. It has always been considered that, in TMD, the IL-6 at the synovial fluid comes from synoviocytes, chondrocytes, or inflammatory cells as the main source. But, depending on the TMD-type, masticatory muscles should be a new source to keep in mind, considering its great contribution to the biomass of the system.

## Muscle-Bone Crosstalk at the Masticatory System in Health and Disease—Moving to Clinical Evidence

In the present section, we discuss clinical data suggesting a biochemical communication between muscle and bone at the masticatory system. To this end, we reviewed the evidence on several muscular pathologies or clinical interventions leading to bone remodeling, as well as bone pathologies leading to muscle remodeling. We focus on the presence of molecules described in the previous sections as myokines or osteokines.

### Muscular Conditions With Potential Bone Implications in the Masticatory System

During prolonged tooth clenching, the masseter muscle exhibits a lower recovery capacity for tissue reoxygenation (176, 177), which favors the development of skeletal muscle inflammation. Britto et al. have demonstrated that hypoxia-evoked inflammation leads to skeletal muscle hypertrophy through the IL‐6/STAT3 pathway in human legs (178). These results may explain the potential mechanism behind the masseter hypertrophy in patients with parafunctional masticatory activity (179, 180). The expression of IL-6 is also increased during other inflammatory conditions of the masticatory system, such as myofascial pain (181), which is part of the group of craniofacial musculoskeletal diseases known as TMDs (182, 183). Increased levels of IL-6 have been reported in masseter muscles of adult women with myofascial pain compared to healthy controls, levels that are even higher during tooth clenching (184). Considering the dual role of IL-6 in bone formation and resorption, is highly possible that muscle-derived IL-6 mediate mandibular bone remodeling observed in TMDs.

Masseter hypertrophy is often associated with parafunctional activities such as bruxism (179), but it also may have an idiopathic background (i.e., benign masseter hypertrophy) (185). In both cases, the aesthetic impairment caused by the increase of masseter volume mass can be solved either using surgical techniques or by inducing muscle atrophy with botulinum toxin type A (BoNTA) (186, 187). The BoNTA is a neurotoxin that blocks the release of neurotransmitters in the skeletal muscle, leading to hypofunction and atrophy (188). Therefore, BoNTA-induced atrophy is effective in reducing the thickness of the masseter muscle (189). This desired aesthetic outcome, however, may involve deleterious consequences on mandibular bone homeostasis (51, 125). In patients that underwent repetitive BoNTA injections to treat masseter hypertrophy, a reduction of bone volume at the mandibular angle was found after 6 months (190). Another study found bone loss in the anterior portion of the mandibular condylar process 1 year after a single injection of BoNTA in both the masseter and the temporalis muscles (191). Using a similar design, a retrospective study identified in adult women a cortical bone thinning in the mandibular condylar processes after two BoNTA injections within a 6-month interval (192). In this context, the BoNTA intervention resulted in a more deleterious effect at the cortical bone of the mandibular condylar process of postmenopausal women, when compared with premenopausal women (192). In adult women with myofascial pain, a randomized clinical trial demonstrated a significant reduction of the volume of the mandibular condylar process at 3 months, after a high BoNTA dose injection in both the temporalis (25 U) and the masseter (75 U) muscles but not at lower doses (20 U and 50 U, respectively) (193). These results are consistent with a cohort study, which showed no bone loss at the mandibular condylar process in adult women with myofascial pain that underwent BoNTA interventions under 40 U in the masseter muscles when compared to match control population (194). These results support the hypothesis that masticatory muscle hypofunction negatively impacts mandibular bone homeostasis in humans, especially at the condylar process, when high doses of BoNTA are used. Based on our results in a mouse model, it is advisable to characterize how the negative effect of BoNTA-induced masseter atrophy on the mandibular bone occurs. Is it limited to a biomechanical interaction, or does it respond to alteration on the secretory activity of soluble factors from the injected muscles, such as IL-6? The answer to this question may help to develop strategies to avoid BoNTA’s deleterious effect on the mandible.

### Bone Conditions With Potential Muscle Implications in the Masticatory System

As mentioned above, the jaws undergo pathologies that are specific to them, but they are also affected by more general conditions such as bone loss during aging (195). Since bone also works as an endocrine organ (12), pathological conditions that affect the capacity of the mandibular bone to release osteokines could also affect muscle-bone molecular crosstalk. Here we analyze how mandible-changes could lead to masticatory muscles remodeling during aging, microgravity, and periodontal disease in humans.

In postmenopausal women, a lower mandibular bone density and higher plasma levels of osteocalcin were determined when compared with premenopausal women (195). These results found as a consequence of aging are consistent with those identified during space flight conditions (i.e., microgravity) (196). In adults of both sexes, the use of simulated microgravity promotes a reduction of bone mineral density in the mandibular bone and an increase in the plasma and salivary levels of osteocalcin (197). Interestingly, in an animal study under microgravity conditions, the masticatory muscles were not atrophied, in contrast with those from the hindlimbs (198). The fact that the activity of the masticatory muscles seems not to be affected by the lack of gravity may shed light on their structural and physiological differences to postcranial muscles.

Osteocalcin has been linked to muscle hypertrophy (12). In a mouse model of forceful mastication, an increase of osteoblasts positive for osteocalcin in the enthesis between the masseter and mandibular bone was observed (132). Even more, recently has been described that a muscle-bone axis comprising IL-6 released by muscles and osteocalcin released and processed by osteoblasts/osteoclasts is relevant to improve muscle performance during exercise (80). It seems contradictory, then, to observe increased plasma levels of osteocalcin in older adults and hypogravity models, which present a clear decrease in muscle mass in the trunk and limbs. One possibility is that, since osteocalcin is regulating the levels of IL-6 secreted by the muscle, it may also promote the adverse catabolic effects of IL-6 on muscles and bones. Probably a fine-tuned muscle-bone axis is controlling anabolic or catabolic final effects, depending on myokines/osteokines concentrations, or modulated by additional microenvironment stimuli. It is still unknown how these molecular pathways differentially affect masticatory versus trunk and limbs musculoskeletal system. The existence of features that appear contradictory, such as bone loss with an increased osteocalcin production and a preserved muscle activity, presents an exciting opportunity to investigate the functional peculiarities of the musculoskeletal components of the masticatory apparatus.

In addition, while mandibular osteocytes are activated during high load masticatory activity (132), the aging process seems to affect their number only in the bones of the hindlimbs but not in those of the craniofacial skeleton, including the mandible (199, 200). *In vitro*, it has been shown that osteocytes also produce osteokines that impair skeletal muscle homeostasis (12, 201). One of these osteocyte-derived osteokines is sclerostin (202). Since masticatory function suppresses the release of sclerostin from mandibular osteocytes (132), a reduced masticatory function during aging increases the levels of this osteokine, which may impact the masticatory muscles negatively through a molecular (and not purely mechanical) mechanism.

The inflammatory periodontal disease that causes alveolar bone loss (i.e., periodontitis) increases the level of osteokines like osteocalcin in the gingival crevicular fluid (GCF) of postmenopausal women (203). This increase, however, was not found in the saliva or plasma of the periodontally ill patients (203). Moreover, there is a lack of correlation between the presence of systemic bone disease (osteopenia and osteoporosis) and osteocalcin levels in either salivary, plasma or GCF samples (203–205). Another recognized osteokine, sclerostin, is also increased in the GCF of periodontally ill patients (12, 206). In bone tissue, sclerostin is a negative regulator of bone mass through the inhibition of the Wnt signaling in the osteoblast lineage (206). The osteoblast population of the human mandible, however, exhibits a higher Wnt signaling response to external vibration when compared with osteoblast from the iliac bone (207). In adults with moderate to severe periodontitis, a significant increase of sclerostin in the GCF was determined when compared with healthy patients (208). In contrast, Wnt proteins levels in the GCF were no significatively different between periodontally ill and healthy patients but were increased in individual sampled sites (periodontally compromised) when compared with healthy sites (208). Taken together, the results of this clinical study suggest both sclerostin and Wnt proteins as a promising diagnostic tool for periodontitis (208). Since sclerostin has been presented as an osteokine with catabolic potential on muscle cells, these results allow us to hypothesize a molecular (and not purely mechanical) link between periodontitis and the reduction of the masticatory muscle thickness that has been found in periodontally ill patients (209).

The osteocyte population is a crucial regulator of both sclerostin and RANKL local expression during active periodontal disease (210). The sclerostin and RANKL are negative bone mass regulators by inhibiting Wnt signaling and by inducing osteoclastogenesis, respectively (210–212). Specifically, the receptor of RANKL, RANK, is expressed in the skeletal muscle tissue (213) and a deleterious effect of RANKL on muscle homeostasis has been suggested (212, 214). Although periodontitis is known to increase the systemic inflammatory burden affecting, e.g., the cardiovascular system (215), it is reasonable to hypothesize that inflammatory diseases of the jaws can affect masticatory muscle homeostasis, as these are anatomically closer and linked through the vascular network.

## Concluding Remarks and Further Perspectives

In this review, we summarized and discussed the available information regarding the muscle-bone interaction in the masticatory apparatus, with an emphasis in the molecular crosstalk between both tissues, an emerging research area that shows promising applications in clinics. The structures of the masticatory apparatus present biochemical, structural, and functional characteristics that make them physiologically very different from the musculoskeletal components of the trunk and limbs. The bones in the masticatory apparatus also have a high rate of remodeling, not only during development and postnatal growth but well into adulthood. In addition, an essential morphofunctional relationship between the muscles and bones has been described in this region.

To date, the approach to study the muscle-bone crosstalk in the masticatory apparatus has been mostly biomechanical. Here, we present the evidence suggesting that the communication between the jaws and masticatory muscles also occurs *via* secreted molecules, which opens a new field of research. Molecules defined as “myokines” (e.g., IGF-1 and IL-6) or “osteokines” (eg, Osteocalcin, Sclerostin, RANKL) have been described as expressed in the masticatory apparatus. The levels of these mediators are altered both in animal models of use/disuse of the masticatory apparatus, as well as in pathophysiological conditions in humans. Due to the large biomass component provided by the masticatory muscles, it is highly probable that they contribute through myokines in the pathogenesis of temporomandibular disorders. Molecules such as IL-6, which have been reported elevated in the synovial fluid of individuals affected by TMDs, and have been essentially associated with chondrocytes or inflammatory cells, could well be derived from masticatory muscles. Likewise, the molecules that mediate bone resorption associated with periodontitis could cross-affect masticatory muscles and contribute to the loss in their volume.

It is already challenging to experimentally separate the biomechanical from the biochemical component in the musculoskeletal system, but even more so in the masticatory apparatus, which due to its structural characteristics makes it difficult to work with isolated cells. For example, the culture of isolated fibers is of common use to study limb muscles. It is, however, very complex in multipennate muscles such as the masseter, with fibers of very different lengths, orientations, and firmly attached to bone and tendons. To our knowledge, obtaining isolated masseter muscle fibers has been briefly described in only one article (216), but to date, no cellular or biochemical studies have been reported that use them *in vitro*. Likewise, obtaining bone precursors for *in vitro* cultures, which is easy from long bones such as the femur or the tibia, is operationally much more challenging in the mandible (217). It therefore remains a challenge to find experimental designs that allow for evaluating the biochemical muscle-bone crosstalk in the masticatory apparatus. Probably, genetic manipulation approaches, directed to proteins in specific cell types, will be relevant in this mission.

The understanding of how the cells of the masticatory apparatus (muscle, bone, cartilage, immune) communicate through molecules, both in health and disease, will contribute to the global understanding of how the masticatory apparatus remodels. More importantly, it will allow for having precise therapeutic targets, focused not only to alleviate the symptoms but to tackle some prevalent orofacial pathologies from their bases.

## Author Contributions

SB proposed the concept of the review. SB, JB-M, KK, and VT-I wrote and edited the manuscript. WV and CB contributed new data for the topic discussion. SB, JB-M, and VT-I designed the figures. All authors contributed to the article and approved the submitted version.

## Funding

This work was financed by Fondecyt-Chile Grant N°1201385 (SB, VT-I, KK, JB-M) and Universidad de Chile VID-Enlace Fondecyt 2019 Grant N° ENL29-19 (SB).

## Conflict of Interest

The authors declare that the research was conducted in the absence of any commercial or financial relationships that could be construed as a potential conflict of interest.

The handling editor declared a past co-authorship with the authors SB, JB-M.
